# Supplementary material for: A physically inspired approach to coarse-graining transcriptomes reveals the dynamics of aging
Source: PLoS One. 2024 Oct 29;19(10):e0301159. doi: 10.1371/journal.pone.0301159 (PMC11521254; doi:10.1371/journal.pone.0301159)
Supplement: S1 Appendix — (PDF) [file pone.0301159.s001.pdf]

S1 Appendix. Quality Control This section discusses the importance of performing quality control on the raw data before normalization. It explains the specific approach used for the Tabula Muris Senis data, which involves screening out poorly sequenced genes expressed in less than 5% of cells. The rationale behind this threshold is provided, considering the remaining genome size and normalization performance.

For the Tabula Muris Senis data, the empirical choice was to screen out genes expressed in less than 5% of sequenced cells. This threshold was chosen based on two considerations:

1. **Remaining Genome Size:** It is undesirable to screen out too many genes, as it may hinder downstream analysis. At the 5% threshold, approximately 60% of the genome is retained for all cell types. If the threshold is increased to 20%, the remaining genome size would be overly reduced to $100 \sim 1000$.

2. **Normalization Performance:** In terms of variance stabilization, the 5% threshold yields normalized counts with the variance of gene counts being well stabilized around 1.

When studying aging dynamics, it is crucial to analyze the same gene set that is aging to investigate the progression of correlation structures. To achieve this, the quality control process should yield the same gene set across different age groups. This was accomplished by screening out the union of poorly read genes for the three age groups simultaneously, allowing for the discussion of "aging" of the entire genome.
