# Supplementary material for: A physically inspired approach to coarse-graining transcriptomes reveals the dynamics of aging
Source: PLoS One. 2024 Oct 29;19(10):e0301159. doi: 10.1371/journal.pone.0301159 (PMC11521254; doi:10.1371/journal.pone.0301159)
Supplement: S2 Appendix — (PDF) [file pone.0301159.s002.pdf]

S2 Appendix & Fig. Data Normalization The normalization method used in this work is analytic Pearson residuals [15]. It is based on a common modeling assumption for count data without biological variability. Assume each gene g takes up a fraction p_g of the total amount n_c of counts in cell c. The read counts are then modeled as Poisson or negative binomial samples with expected value $\mu_{cg} = p_g n_c$ without zero-inflation:

$$X_{cg} \sim Poisson(\mu_{cg}) \text{ or } NB(\mu_{cg}, \theta) \tag{1}$$

where θ is a dispersion parameter. The expected value of a gene count is then divided into two parts: gene-specific effect and cell-specific effect, $\mu_{cg} = n_c p_g$. In the Poisson model, the MLE solution is exact: $\hat{n}_c = \sum_g X_{cg}$ (sequencing depth) and $\hat{p}_g = \sum_c X_{cg} / \sum_c \hat{n}_c$. Or equivalently,

$$\hat{\mu}_{cg} = \frac{\sum_j X_{cj} \sum_i X_{ig}}{\sum_{ij} X_{ij}} \tag{2}$$

which proves to be a good approximation of MLE solution for negative binomial model. Then the normalized counts are given by:

$$Z_{cg} = \frac{X_{cg} - \hat{\mu}_{cg}}{\sqrt{\hat{\mu}_{cg} + \hat{\mu}_{cg}^2/\theta}} \tag{3}$$

where the denominator $\sqrt{\hat{\mu}_{cg} + \hat{\mu}_{cg}^2/\theta}$ is the approximated NB variance. We use he most common practice of the choice with $\theta = 50$.

This normalization method restores the gene counts into their analytical z-score, which is assumed to have zero mean and unit variance (Fig S1).
