# Supplementary material for: A physically inspired approach to coarse-graining transcriptomes reveals the dynamics of aging
Source: PLoS One. 2024 Oct 29;19(10):e0301159. doi: 10.1371/journal.pone.0301159 (PMC11521254; doi:10.1371/journal.pone.0301159)
Supplement: S3 Appendix — (PDF) [file pone.0301159.s003.pdf]

S3 Appendix & Fig. Null Analysis In the empirical setting, a null analysis seems appropriate. As prescribed in the main text, a null data is generated by marginal resampling. To be more specific, a gene expression data is an N-by$M$ matrix, where N is the number of genes and M is the number of sequenced cell. By marginal resampling, we simply shuffle each row of the gene expression matrix. That is, the individual distribution of every single gene is preserved. However, by such a resampling, the correlation structures no longer exist, giving us an appropriate null model to investigate.

Fig S2 demonstrates both the coarse-graining methods applied to the null data. In panal (a.ii) and (b.ii), we can clearly see that both coarse-graining methods eventually converge to a standard normal distribution. (a.i) further confirms the fact. The block structure in correlation matrices would also never appear since any correlations have been destroyed by the marginal resampling. So the eigenvalue spectrum would be nearly continuous and flat as predicted by Marchenko-Pasture distribution and the corresponding spectral gap would be super small.
