# Supplementary material for: A physically inspired approach to coarse-graining transcriptomes reveals the dynamics of aging
Source: PLoS One. 2024 Oct 29;19(10):e0301159. doi: 10.1371/journal.pone.0301159 (PMC11521254; doi:10.1371/journal.pone.0301159)
Supplement: S4 Appendix — (PDF) [file pone.0301159.s004.pdf]

S4 Appendix. Usage of Quantitative Analysis.

Spectral Gap We used spectral gap to assess the block structure in correlation matrices. It can be easily related to the block structure by principal component analysis. In traditional PCA, a block in the correlation matrix will correspond to a large-variance principal component. Suppose two correlation matrices have similar block structures. The spectral gap among blocks or between the smallest block and other modes is maximized when all the blocks are uncorrelated. That being said, the block structures are outstanding. Otherwise, if there are many interwinding correlations among blocks, the spectral gap will shrink. Eventually, when the block structure vanishes, the eigenvalue spectrum is expected to be continuous so the spectral gap would be very small.

Anderson-Darling Test The formal definition of the Anderson-Darling normality statistics [17] for an empirical set $\{Y_i\}$ is

$$A^2 = -M - \frac{1}{M}\sum_{i=1}^{M}(2i-1)\left[\ln\Phi(Y_i) + \ln(1-\Phi(Y_i))\right] \tag{4}$$

where Φ is the standard normal cumulative distribution function. As suggested by D'Agostino (1986), the statistics should be adjusted as

$$A_*^2 = A^2\left(1 + \frac{3}{4n} + \frac{9}{4n^2}\right) \tag{5}$$

Apparently, the Anderson-Darling is associated with the sample size. In order to eliminate the effect of different sample size when we compare the normality across different groups, we performed bootstrap. More specifically, since the number of sequenced cells are 3,000 for each group, we use bootstrap to resample 6,000 cells for every single gene when calculating the Anderson-Darling normality statistics.
