# Supplementary material for: A physically inspired approach to coarse-graining transcriptomes reveals the dynamics of aging
Source: PLoS One. 2024 Oct 29;19(10):e0301159. doi: 10.1371/journal.pone.0301159 (PMC11521254; doi:10.1371/journal.pone.0301159)
Supplement: S5 Appendix — (PDF) [file pone.0301159.s005.pdf]

S5 Appendix. Data Availibility Tabula Muris Senis data is a high-through RNA-sequencing data of mice for different ages that was developed by Tabula Muris team. The data is currently available at figshare and more information can be found in the original paper: https://www.nature.com/articles/s41586-020-2496-1.
