# Supplementary figures and images for: A physically inspired approach to coarse-graining transcriptomes reveals the dynamics of aging
Source: PLoS One. 2024 Oct 29;19(10):e0301159. doi: 10.1371/journal.pone.0301159 (PMC11521254; doi:10.1371/journal.pone.0301159)

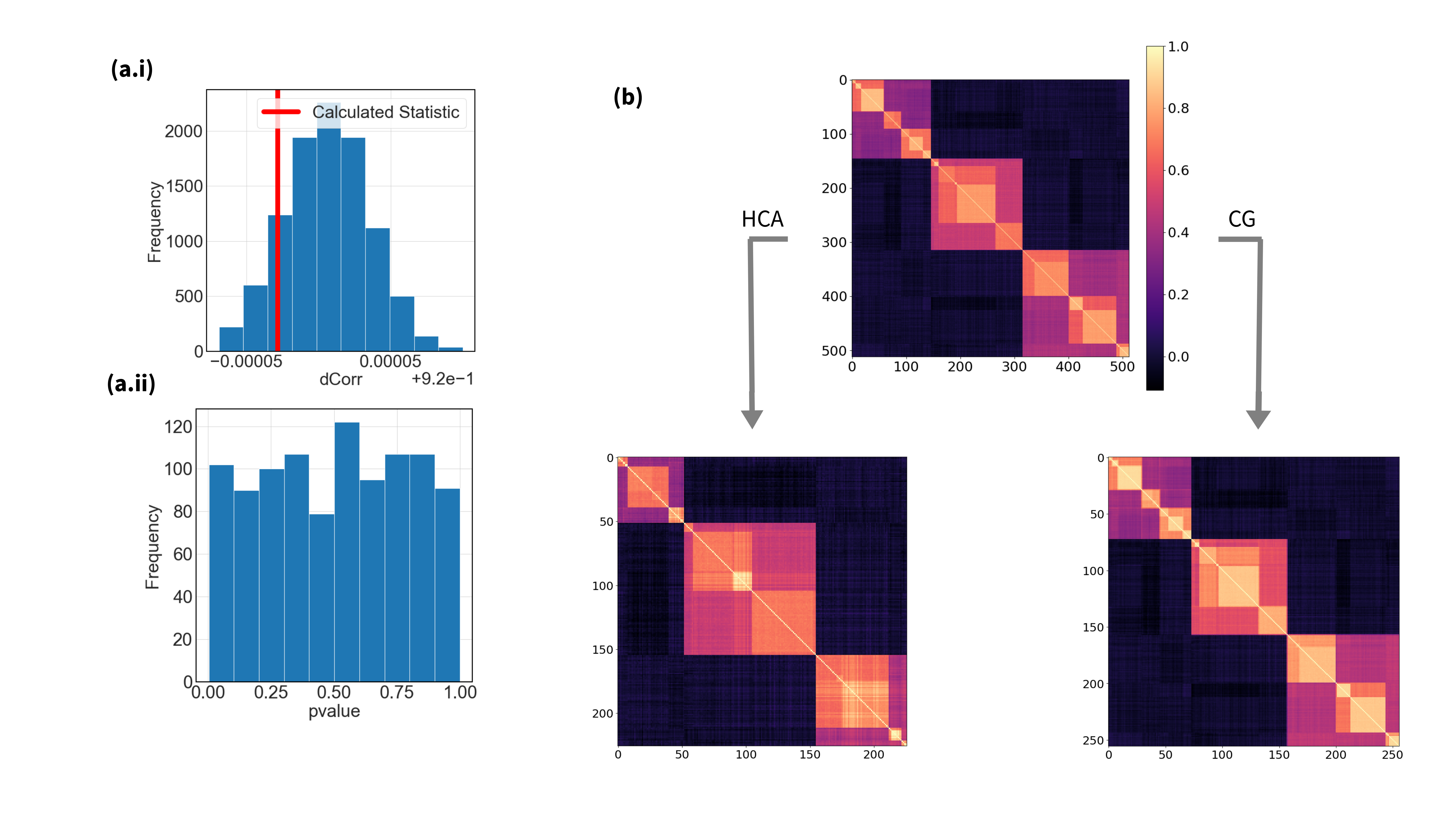

Supplement: S3 Fig — (a): Validation of the proposed bootstrapping test, obtained from two random matrices that have different size. (a.i): The dCorr distribution from one bootstrapping simulation. (a.ii): The p-value distribution from 1,000 bootstrapping simulation. (b): (Top) The correlation matrix of toy model. (Bottom) Two resulting correlation matrices after 1 step of aggregating and averaging for both methods. (TIFF) [file pone.0301159.s008.tiff]
