# Supplementary material for: A physically inspired approach to coarse-graining transcriptomes reveals the dynamics of aging
Source: PLoS One. 2024 Oct 29;19(10):e0301159. doi: 10.1371/journal.pone.0301159 (PMC11521254; doi:10.1371/journal.pone.0301159)
Supplement: S1 Table — (PDF) [file pone.0301159.s009.pdf]

	HCA	CG
Max *p*-value	1e-3	1e-9
Avg. Null dCorr	0.77	0.79
Avg. Real dCorr	0.87	0.97

Table S1. The bootstrapping statistics
